# *Phialolunulospora vermispora* (Chaetosphaeriaceae, Sordariomycetes), a novel asexual genus and species from freshwater in southern China

**DOI:** 10.3897/mycokeys.76.57410

**Published:** 2020-12-22

**Authors:** Hua Zheng, Yake Wan*, Jie Li, Rafael F. Castañeda-Ruiz, Zefen Yu

**Affiliations:** 1 Laboratory for Conservation and Utilization of Bio-resources, Key Laboratory for Microbial Resources of the Ministry of Education, School of Life Sciences, Yunnan University, Kunming, Yunnan, 650091, China Yunnan University Kunming China; 2 Instituto de Investigaciones Fundamentales en Agricultura Tropical “Alejandro de Humboldt” (INIFAT), 17200, La Habana, Cuba Instituto de Investigaciones Fundamentales en Agricultura Tropical “Alejandro de Humboldt” La Habana Cuba

**Keywords:** Biodiversity, Chaetosphaeriales, phylogeny, taxonomy

## Abstract

The asexual taxon *Phialolunulospora vermispora***gen. et sp. nov.**, collected from submerged dicotyledonous leaves in Hainan, China, is described and illustrated herein. *Phialolunulospora***gen. nov.** is characterized by macronematous, semimacronematous, septate and pigmented conidiophores and acrogenous, long lunate, vermiform to sigmoid, hyaline conidia with an eccentric basal appendage. Complete sequences of internal transcribed spacer (ITS) and partial sequences of nuclear large subunits ribosomal DNA (LSU) genes are provided. Phylogenetic analyses of combined ITS and LSU sequences revealed its placement in the Chaetosphaeriaceae. The new fungus is compared with morphologically similar genera.

## Introduction

China is considered an important Asian reservoir of biodiversity. The southern area of China ranks 34^th^ in biodiversity hotspots (Myers et al. 2000; Williams et al. 2001). Hainan Island, located in the south of China, harbors an incredibly high diversity of fungi. Its humid, subtropical climate, with an average annual temperature of 22 to 27 °C and an average annual precipitation of 1000–2600 mm, favors development of fungi. Our group has conducted investigations of freshwater fungi to increase knowledge of this important ecological group in China (Qiao et al. 2017a, b, 2018a, b, 2019a, b, 2020).

During our present investigation of freshwater fungi in Hainan Island, South China, an interesting species was collected on dead leaves of an unidentified dicotyledonous tree. This species is characterized by unbranched and septate conidiophores, phialidic conidiogenous cells and vermiform to sigmoid and aseptate conidia with an eccentric basal appendage. Based on preliminary analysis of morphological data, we place this unknown fungus in Chaetosphaeriaceae, but a literature search found that it did not belong to any known genus. To further confirm the position of the species, phylogenetic analyses with related taxa within Chaetosphaeriaceae were carried out based on complete sequences of internal transcribed spacer (ITS) and partial sequences of nuclear large subunits ribosomal DNA (LSU) genes.

## Materials and methods

### Isolation and morphological study

Submerged dicotyledonous leaves were collected from Limu Mountain Nature Reserve in Hainan Province. Samples were preserved in zip-lock plastic bags, labelled, and transported to the laboratory. The decomposed leaves were cut into several 2–4 × 2–4 cm sized fragments and then spread on to the surface of corn meal agar (CMA, 20 g cornmeal, 18 g agar, 40 mg streptomycin, 30 mg ampicillin, 1000 ml distilled water) medium for 10 days; single conidium was isolated with a sterilized needle and transferred to CMA plates while viewing with an Olympus BX51 microscope. The pure strain was further transferred to potato dextrose agar (PDA, 200 g potato, 20 g dextrose, 18 g agar, 40 mg streptomycin, 30 mg ampicillin, 1000 ml distilled water) medium. Colony morphology and microscopic characteristics were examined, and photographs were taken with an Olympus BX51 microscope connected to a DP controller digital camera. Measurement data were based on 30 random conidia and 10 conidiophores.

Pure cultures were deposited in the Herbarium of the Laboratory for Conservation and Utilization of Bio resources, Yunnan University, Kunming, Yunnan, China (**YMF**, formerly Key Laboratory of Industrial Microbiology and Fermentation Technology of Yunnan) and at the China General Microbiological Culture Collection Center (**CGMCC**).

### DNA extraction, PCR amplification, and sequencing

Pure cultures were grown on PDA medium for 5 days at 25 °C. Actively growing mycelium was scraped off from the surface of the culture and transferred to 2 ml Eppendorf micro-centrifuge tubes. Total genomic DNA was extracted according to the procedures in Turner et al. (1997). Primers used for PCR amplification and sequencing of the nuclear large subunits ribosomal DNA (LSU) and the internal transcribed spacer (ITS) were LROR-LR7 and ITS1-ITS4, respectively (Vilgalys and Hester 1990; White et al. 1990). PCR products were purified and stored at -20 °C until sequencing. The same pairs of primers were used to obtain sequences, which was performed by Macrogen Europe (Macrogen Inc. Amsterdam, The Netherlands). Finally, the sequences were assembled and edited using SeqMan v. 7.0.0 (DNAStar Lasergene, Madison, WI, USA) to obtain the consensus sequences. The newly obtained sequences were submitted to GenBank nucleotide database (Table 1).

**Table 1.** List of strains analyzed in this study, with GenBank accession numbers.

Species	Strain	ITS	LSU
*Adautomilanezia caesalpiniae*	LAMIC 010212	NR_153560	NG_058594
*Anacacumisporium appendiculatum*	HMAS 245593^T^	KT001555	KT001553
*Anacacumisporium appendiculatum*	HMAS 245602	KT001556	KT001554
*Bahusutrabeeja dwaya*	CBS 261.77^T^	MH861059	MH872829
*Brunneodinemasporium brasiliense*	CBS 112007^T^	JQ889272	JQ889288
*Brunneodinemasporium jonesii*	GZCC 16–0050^T^	KY026058	KY026055
*Cacumisporium capitulatum*	FMR 11339	HF677176	HF677190
*Cacumisporium capitulatum*	SMH 3766	–	AY017374
*Calvolachnella guaviyunis*	CBS 134695	NR_153892	NG_058879
*Chaetosphaeria ciliata*	CBS 122131^T^	MH863180	MH874726
*Chaetosphaeria ciliata*	ICMP 18253	–	GU180637
*Chloridium chloroconium*	FMR 11940	KY853435	KY853495
*Chloridium* sp.	HGUP 1806	MK372070	MK372068
*Codinaea lambertiae*	CBS 143419^T^	NR_156389	NG_059053
*Codinaea pini*	CBS 138866^T^	NR_137943	NG_058902
*Conicomyces pseudotransvaalensis*	HHUF 29956^T^	NR_138015	LC001708
*Cryptophiale hamulata*	MFLUCC 180098	–	MG386756
*Cryptophiale udagawae*	MFLUCC 180422	MH758198	MH758211
*Cryptophialoidea fasciculata*	MFLUCC 172119	MH758195	MH758208
*Dendrophoma cytisporoides*	CBS 223.95^T^	JQ889273	JQ889289
*Dictyochaeta ellipsoidea*	MFLUCC 181574^T^	MK828628	MK835828
*Dictyochaeta lignicola*	DLUCC 0899^T^	MK828630	MK835830
*Dictyochaeta assamica*	CBS 242.66	MH858788	MH870426
*Dictyochaetopsis gonytrichoides*	CBS 593.93	AF178556	AF178556
*Dinemasporium morbidum*	CBS 129.66^T^	JQ889280	JQ889296
*Dinemasporium polygonum*	CBS 516.95^T^	NR_137786	NG_059109
*Echinosphaeria canescens*	SMH 4791	–	AY436403
*Eucalyptostroma eucalypti*	CBS 142074^T^	NR_154027	NG_059257
*Eucalyptostroma eucalyptorum*	CPC 31800^T^	NR_159834	MH327838
*Exserticlava vasiformis*	TAMA 450	–	AB753846
*Gelasinospora tetrasperma*	CBS 178.33	NR_077163	DQ470980
*Helminthosphaeria clavariarum*	SMH 4609^T^	–	AY346283
*Infundibulomyces cupulata*	BCC 11929^T^	EF113976	EF113979
*Infundibulomyces oblongisporus*	BCC 13400^T^	EF113977	EF113980
*Kionochaeta castaneae*	GZCC 18–0025^T^	MN104610	MN104621
*Kionochaeta microspora*	GZCC 18–0036^T^	MN104607	MN104618
*Lasiosphaeria ovina*	SMH 4605	AY587923	AY436413
*Lecythothecium duriligni*	CBS 101317	–	AF261071
*Leptosporella arengae*	MFLUCC 150330^T^	MG272255	MG272246
*Leptosporella gregaria*	SMH 4290^T^	–	AY346290
*Linocarpon arengae*	MFLUCC 150331^T^	–	MG272247
*Linocarpon cocois*	MFLUCC 150812^T^	MG272257	MG272248
*Menispora glauca*	FMR 12089	HF678528	HF678538
*Menispora tortuosa*	DAOM 231154	KT225527	AY544682
*Menispora tortuosa*	CBS 214.56	AF178558	AF178558
*Menisporopsis breviseta*	GZCC 18–0071^T^	MN104612	MN104623
*Menisporopsis dushanensis*	GZCC 18–0084^T^	MN104615	MN104626
*Morrisiella indica*	HKUCC 10827	–	DQ408578
*Multiguttulispora sympodialis*	MFLUCC 180153^T^	MN104606	MN104617
*Nawawia filiformis*	MFLUCC 160853	–	MH758206
*Nawawia filiformis*	MFLUCC 172394	MH758196	MH758209
*Neonawawia malaysiana*	CBS 125544^T^	GU229886	GU229887
*Paliphora intermedia*	CBS 896.97^T^	NR_160203	NG_057766
*Paliphora intermedia*	CBS 199.95	–	EF204500
*Phaeostalagmus cyclosporus*	CBS 663.70	MH859892	MH871680
*Phaeostalagmus cyclosporus*	CBS 312.75	–	MH872661
***Phialolunulospora vermispora***	YMF 1.04260^T^	**MK165444**	**MK165442**
*Phialosporostilbe scutiformis*	MFLUCC 170227^T^	MH758194	MH758207
*Phialosporostilbe scutiformis*	MFLUCC 181288	MH758199	MH758212
*Pseudodinemasporium fabiforme*	MAFF 244361^T^	AB934068	AB934044
*Pseudolachnea fraxini*	CBS 113701^T^	JQ889287	JQ889301
*Pseudolachnea hispidula*	MAFF 244364	AB934071	AB934047
*Pseudolachnella longiciliata*	HHUF 29962	AB934081	AB934057
*Pseudolachnella yakushimensis*	HHUF 29683^T^	AB934087	AB934063
*Pseudolachnella pachyderma*	HHUF 29955	AB934085	AB934061
*Pyrigemmula aurantiaca*	CBS 126743^T^	HM241692	HM241692
*Pyrigemmula aurantiaca*	CBS 126744	HM241693	HM241693
*Rattania setulifera*	GUFCC 15501	GU191794	HM171322
*Ruzenia spermoides*	SMH 4606	–	AY436422
*Sordaria fimicola*	CBS 508.50	MH856730	MH868251
*Sporoschisma hemipsilum*	SMH 2125	–	AF466083
*Sporoschisma hemipsilum*	SMH 3251	–	AF466084
*Stanjehughesia vermiculata*	HKUCC 10840	–	DQ408570
*Striatosphaeria codinaeaphora*	MR 1230	AF178546	AF178546
*Striatosphaeria codinaeaphora*	SMH 1524	–	AF466088
*Synaptospora plumbea*	SMH 3962	–	KF765621
*Tainosphaeria jonesii*	GZCC 16–0053	KY026059	KY026056
*Tainosphaeria jonesii*	GZCC 16–0065	KY026060	KY026057
*Tainosphaeria monophialidica*	MFLUCC 180146^T^	–	MN104616
*Thozetella pandanicola*	MFLUCC 160253^T^	MH388366	MH376740
*Thozetella tocklaiensis*	CBS 378.58^T^	MH857817	MH869349
*Verhulstia trisororum*	CBS 143234^T^	MG022181	MG022160
*Zanclospora iberica*	CBS 130426^T^	KY853480	KY853544
*Zanclospora iberica*	FMR 12186	KY853481	KY853545

*Sequences generated in this study are emphasized in bold face. ^T^ex-type cultures.

### Sequence alignment and phylogenetic analysis

Preliminary BLAST searches with the ITS and LSU sequences of our strain against the GenBank nucleotide database determined the closely related species (Altschul et al. 1990). BLAST search showed that our strain has homology to species in Chaetosphaeriaceae. Based on this information, related sequences of the two marker loci, which include 72 representatives belonging to Chaetosphaeriaceae, 4 representatives of Helminthosphaeriaceae, 2 representatives of Linocarpaceae and 2 representatives of Leptosporellaceae, were downloaded according to recent studies (Yang et al. 2016, 2018; Wei et al. 2018; Lin et al. 2019). Sordaria fimicola (Roberge ex Desm.) Ces. & De Not, *Gelasinospora tetrasperma* Dowding and *Lasiosphaeria ovina* (Pers.) Ces. & De Not were used as the outgroup. These, together with the newly generated sequences, were aligned with ClustalX 1.83 (Thompson et al. 1997) with default parameters, and the consensus sequences were manually adjusted and linked through BioEdit v.7.0 (Hall 1999). Manual gap adjustments were done to improve the alignment and ambiguously aligned regions were excluded. Then, the combined alignment was converted to a NEXUS file using the program MEGA6 (Tamura et al. 2013) and a PHY files using the program ClustalX 1.83. The resulting combined sequence matrix included 1475 nucleotide positions (with alignment gaps) from two regions (607 from ITS, 868 from LSU). GenBank accession numbers of downloaded sequences are given in Table 1.

Maximum-likelihood (ML) analysis was computed with RAxML (Stamatakis 2006) with the PHY files generated with CLUSTAL_X version 1.83, using the GTR-GAMMA model. ML bootstrap proportions (MLBPs) were computed with 1000 replicates. Bayesian inference (BI) analysis was conducted with MrBayes version 3.2.2 (Ronquist and Huelsenbeck 2003). The Akaike information criterion (AIC) implemented in jModelTest version 2.0 was used to select the best fit models after likelihood score calculations were done (Posada 2008). The base tree for likelihood calculations was ML-optimized. HKY+I+G was estimated as the best-fit model under the output strategy of the AIC. Metropolis-coupled Markov chain Monte Carlo (MCMCMC) searches were run for 5 000 000 generations, sampling every 500^th^ generation. Two independent analyses with four chains each (one cold and three heated) were run until the average standard deviation of the split frequencies dropped below

0.01. The initial 25% of the generations of MCMC sampling were discarded as burn-in. The refinement of the phylogenetic tree was used for estimating BI posterior probability (BIPP) values. The tree was viewed in FigTree version 1.4 (Rambaut 2012).

## Results

### Phylogenetic analyses

**Figure 1.**
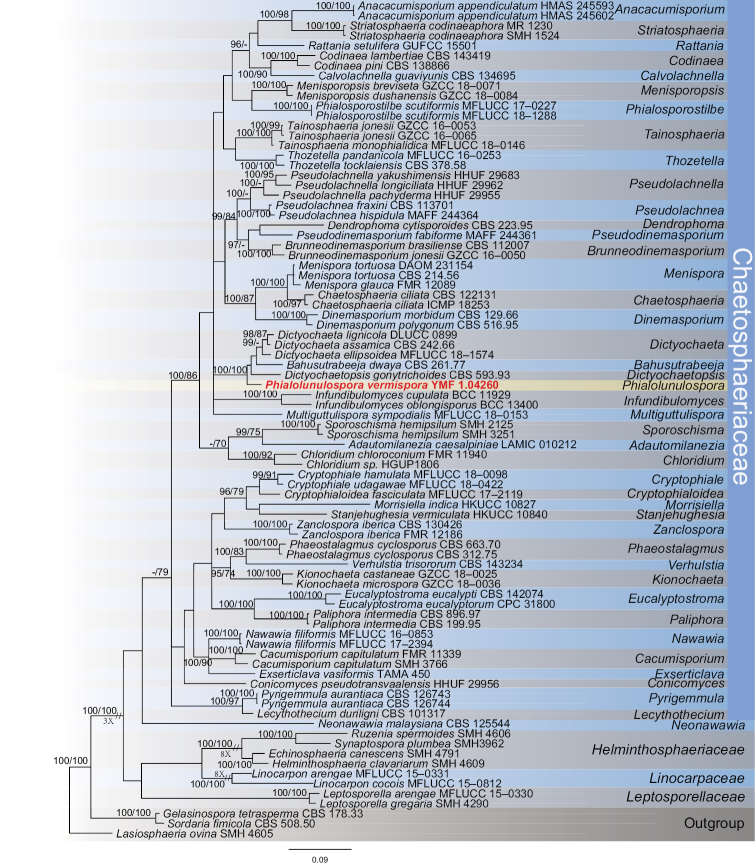
Phylogenetic tree derived from Bayesian analysis based on ITS and LSU sequences, depicting the relationships of the new taxon *Phialolunulospora vermispora* with closely related taxa. The numbers above branches represent BIPP (left) and MLBPs (right). BIPP over 95% and MLBPs greater than 70% are shown on the respective branches, and the bar represents the substitutions per nucleotide position. *Gelasinospora tetrasperma* CBS 178.33, *Sordaria fimicola* CBS 508.50 and *Lasiosphaeria ovina* SMH 4605 were used as outgroup.

The combined dataset comprised 71 taxa (including our strain) representing 52 genera, which include 60 species in the family Chaetosphaeriaceae, 4 species in Helminthosphaeriaceae, 2 species in Linocarpaceae and 2 species in Leptosporellaceae, with *Gelasinospora tetrasperma* CBS 178.33, *Sordaria fimicola* CBS 508.50 and *Lasiosphaeria ovina* SMH 4605 as the outgroup. The final alignment comprised a total of 1475 base pairs, containing the ITS and LSU sequences, and were analyzed by BI and ML method. The topology of the tree is shown in Fig. 1, with the Bayesian posterior probabilities above 95% and ML bootstrap support greater than 70% indicated for respective clades. In this tree, our strain occurred on an isolated clade within Chaetosphaeriaceae, and clustered together with *Dictyochaetopsis* Aramb. & Cabello, *Bahusutrabeeja* Subram. & Bhat and *Dictyochaeta* Speg. with good Bayesian posterior probabilities (100%) and ML bootstrap proportions (100%). Considering distinct morphological characters with these three genera, we propose to describe our unknown isolate as a new genus and species, *Phialolunulospora vermispora*.

### Taxonomy

#### Phialolunulospora

Taxon classificationFungiChaetosphaerialesChaetosphaeriaceae

Z. F. Yu & R. F. Castaneda gen. nov.

C8C22876-17B4-5984-B075-7703241266A8

828716

##### Type species.

*Phialolunulospora vermispora* Z. F, Yu & R. F. Castañeda

##### Etymology.

*Phialo*-Prefix, *Phia. lis* N.L fem. S. Phialide referring to the phialidic conidiogenous cells, and *lunulospora*, (*lu.nu.la.tus* N.L. adj. mean crescent-shaped + *spo.ra* N.L. fem. S. spora, referred to the conidia), referring to the genus *Lunulospora*.

##### Description.

Asexual fungus. *Conidiophores* macronematous, semimacronematous, mononematous, septate, prostrate or erect, straight or flexuous, pigmented. *Conidiogenous cells* integrated, terminal, cylindrical to subulate, pale brown to brown, monophialidic or polyphialidic, enteroblastic. Conidial secession schizolytic. *Conidia* solitary, acrogenous, long lunate, vermiform to sigmoid, unicellular, hyaline, truncate at the conspicuous or inconspicuous basal frill, with a cellular, unbranched, eccentric basal appendage.

#### Phialolunulospora vermispora

Taxon classificationFungiChaetosphaerialesChaetosphaeriaceae

Z. F. Yu & R. F. Castaneda sp. nov.

6A1EB2AB-F23B-54E4-B808-E0AEF505B12D

828717

Figures 2 , 3 , 4

##### Type.

China, Hainan province, Limu Mountain, 19°29'40"N, 107°80'45"E, ca. 350 m alt., from leaves of an unidentified dicotyledonous plant submerged in a stream, Apr 2015, Zefen Yu, YMF 1.04260 – ***holotype***; CGMCC 3.19632 – culture ex-type.

**Figure 2.**
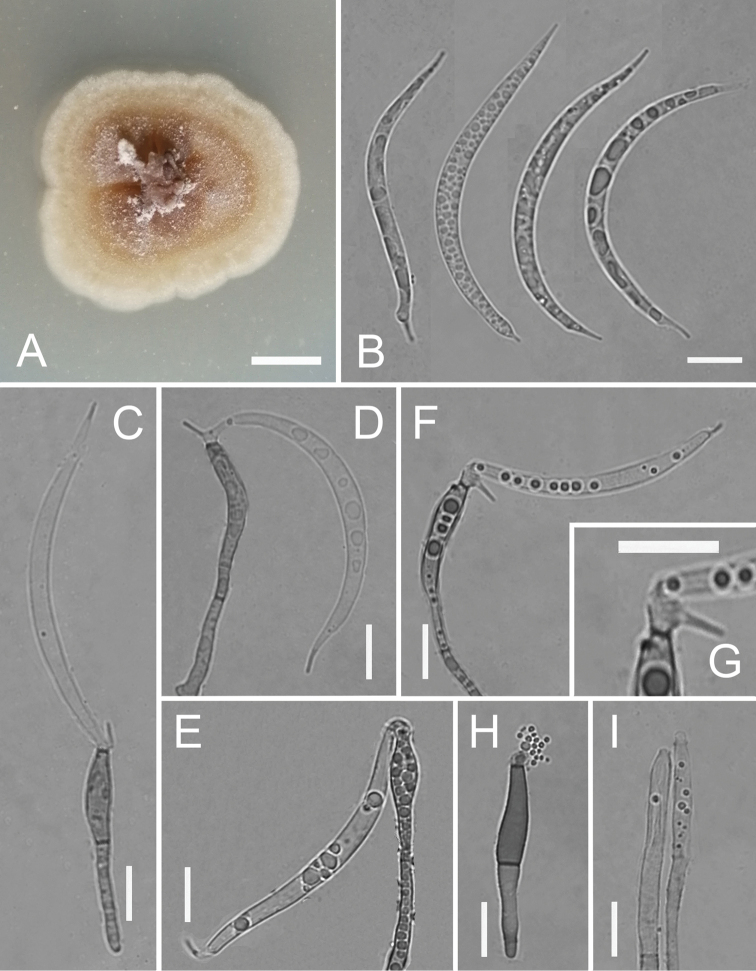
*Phialolunulospora vermispora* (YMF 1.04260) **A** colony on PDA at day 10 **B** conidia **C–F** conidiophores, conidiogenous cells and conidia **G** conidiogenous cells **H, I** conidiophores and conidiogenous cells. Scale bars: 10 mm (**A**); 10 μm (**B–I**).

##### Etymology.

*ver.mi*- (from *vermiformis*), NL fem. adj mean worm-shaped + *spo.ra* N.L. fem. S. spora, referred to worm-shaped conidia.

##### Description.

Mycelium partly superficial and partly immersed, composed of septate, branched, smooth, hyaline, 1–2 μm wide hyphae. *Conidiophores* solitary, macronematous, semimacronematous, erect or prostrate, straight or flexuous, unbranched, up to 4-septate, cylindrical, up to 150 μm long, 3–4 μm wide, pale brown to brown, smooth, sometimes reduced to conidiogenous cells. *Conidiogenous cells* integrated, terminal, cylindrical to subulate, sometimes lageniform, determinate, smooth, pale brown to brown, mostly darker than conidiophores, phialidic, after secession leaving an inconspicuous basal frill, 12–47 × 2.6–3 μm. *Conidia* solitary, acrogenous, long lunate, vermiform to sigmoid, unicellular, guttulate, hyaline, smooth-walled, 31–55 × 2.5–3.5 μm, acute at the apex and narrow truncate at the base bearing minute marginal frills and a cellular, single, unbranched, somewhat attenuated or acuminate, eccentric basal appendage, 1.5–4.6 μm long.

**Figure 3.**
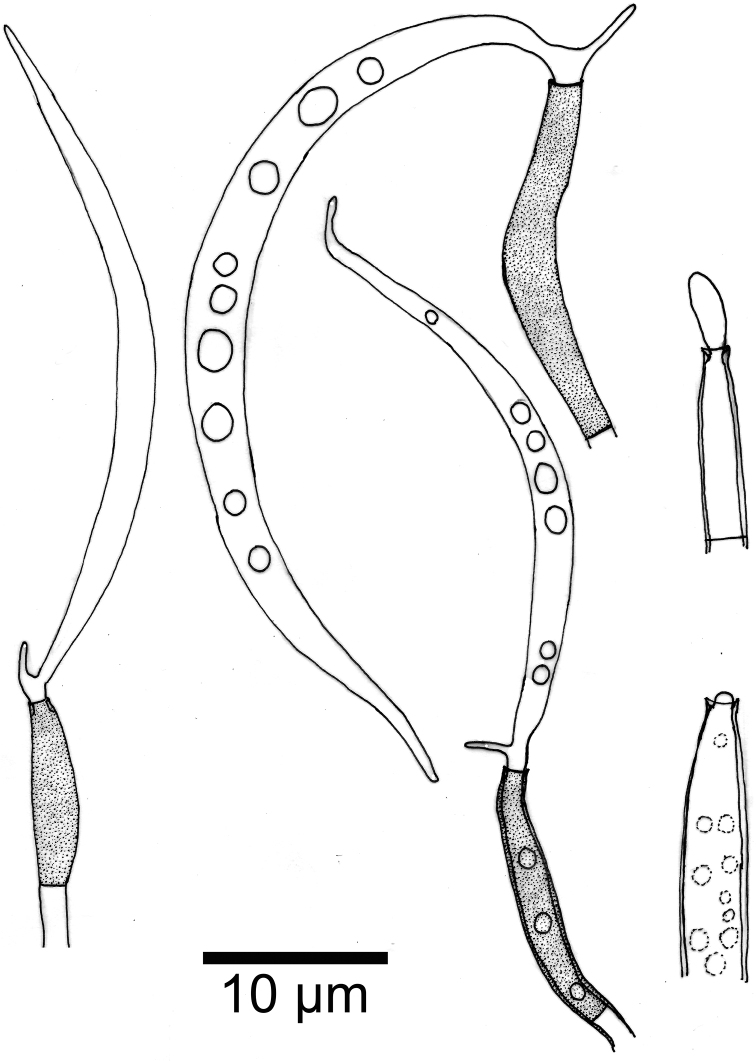
*Phialolunulospora vermispora* conidiogenous cells and conidia.

##### Culture characteristics.

Colonies attain 2.4 cm diameter on PDA and 2.8 cm diameter on CMA after 10 days at 25 °C. On PDA, colonies flat to slightly raised, aerial mycelium abundant, margin entire to undulate, surface white initially, then become buff and grey with age, reverse same color. Colonies on CMA, center with aerial mycelium cottony, periphery with scarce aerial mycelium, olivaceous grey, dark green exudate and soluble pigment produced, reverse same color.

##### Distribution and ecology.

The species occurs on submerged leaves in stream. This species is currently known only from the type locality.

**Figure 4.**
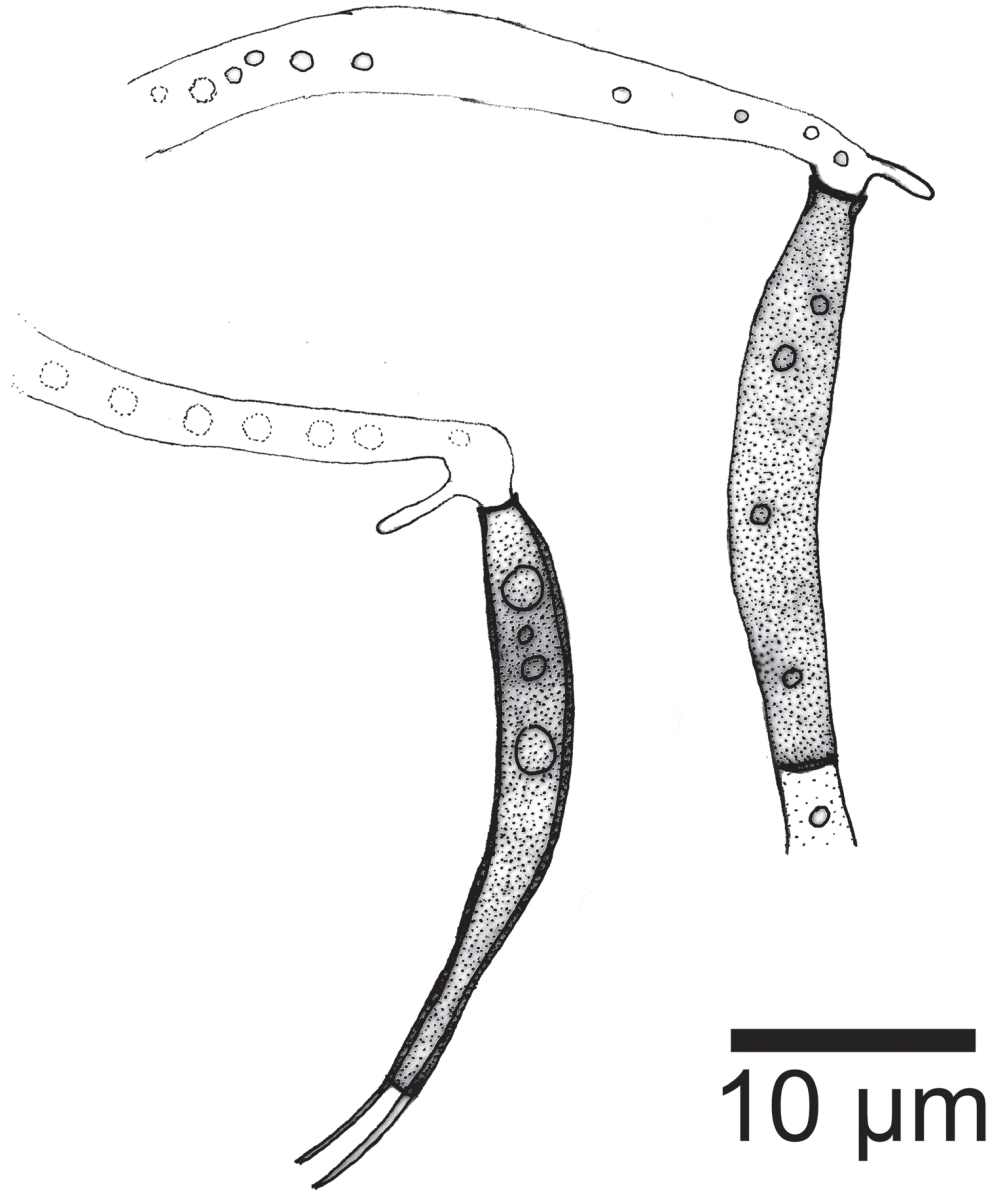
*Phialolunulospora vermispora* conidiogenous cells.

## Discussion

The family Chaetosphaeriaceae was firstly introduced by Réblová et al. (1999) to accommodate *Chaetosphaeria* and its allies. Réblová et al. (1999) also suggested that Chaetosphaeriaceae should be placed in the Sordariales. However, based on the nuclear large subunit ribosomal RNA gene (LSU) sequence, Huhndorf et al. (2004) placed Chaetosphaeriaceae in order Chaetosphaeriales. In a recent review of the family Chaetosphaeriaceae based on morphology and phylogenetic analysis, Lin et al. (2019) accepted 49 genera (including three uncertain genera) within the family, among which 44 were asexual genera.

The asexual morph of the Chaetosphaeriaceae is hyphomycetous taxa. It is characterized by septate, branched or unbranched conidiophores with the conidiogenous cell monophialidic or polyphialidic, holoblastic or enteroblastic, smoothwalled (Réblová et al. 1999, 2011). Our new fungus, *Phialolunulospora vermispora*, fits the general description of asexual hyphomycetous Chaetosphaeriaceae well. *Phialolunulospora* is mainly distinguished from other species in the Chaetosphaeriaceae in having vermiform to sigmoid conidia. Conidia of typical members of the family, including *Dictyochaeta* and *Codinaea* Maire (Réblová 2000; Whitton et al. 2000; Cruz et al. 2008; Crous et al. 2014), are aseptate or 1-septate; they may be setulose or not. In this study, the phylogenetic analyses combining ITS and LSU sequences showed that *P. vermispora* is close to three asexual genera in Chaetosphaeriaceae (Fig. 1), *Dictyochaetopsis*, *Bahusutrabeeja* and *Dictyochaeta*. Morphologically, *Bahusutrabeeja* and *Dictyochaeta* are superficially similar to *P. vermispora* in septate and cylindrical conidiophores, but can be distinguished from the new genus in having globose conidia without appendages and long fusiform conidia with long appendage (Subramanian and Bhat 1977; Li et al. 2014; Liu et al. 2016; Lin et al. 2019), respectively. *P. vermispora* is clearly different from *Dictyochaetopsis* species in morphology, such as smooth and pale brown or brown conidiophores and long lunate, vermiform to sigmoid conidia (Arambarri and Cabello 1990; Whitton et al. 2000; Castañeda-Ruíz et al. 2008).

*Phialolunulospora* is morphologically similar to some other genera species of Chaetosphaeriaceae in hyaline conidia with basal eccentric cellular appendages, including *Neopseudolachnella* A. Hashim. & Kaz. Tanaka, *Pseudolachnea* Ranoj., *Pseudolachnella* Teng and *Rattania* Prabhug. & Bhat. Of these, species of *Neopseudolachnella*, *Pseudolachnea* and *Pseudolachnella* are different from *Phialolunulospora* in acervular, setose and stromatic conidiomata (Ranojevic 1910; Teng and Ling 1936; Hashimoto et al. 2015). The genus *Rattania* is distinguished from *Phialolunulospora* in having seta and smaller septate conidia (Prabhugaonkar and Bhat 2009). In addition, the type species of *Lunulospora* Ingold, *L. curvula* Ingold (Sordariomycetes, Sordariales incertae sedis), also has resemblance to *Phialolunulospora* in conidial shape (Ingold 1942; Seifert et al. 2011), but it has obviously bigger size of conidia, 70–90 × 4–5 μm vs. 12–47 × 2.6–3 μm, in *Lunulospora*.

Many freshwater species occur in the family Chaetosphaeriaceae. So far, approximately 16 genera in this family have been reported from fresh water, such as *Codinaea* (Luo et al. 2019). In this study, *Phialolunulospora vermispora* was also collected from freshwater habitats.

## Supplementary Material

XML Treatment for Phialolunulospora

XML Treatment for Phialolunulospora vermispora
